# International Professional Practice Standards for Clinical Exercise Physiology: Consensus Statement

**DOI:** 10.1007/s40279-026-02407-6

**Published:** 2026-03-25

**Authors:** Nathan E. Reeves, Kirstin N. Lane, Andrew Scott, Kade Davison

**Affiliations:** 1 School of Allied Health, Sport and Social Work, Griffith University, Gold Coast, QLD 4222 Australia; 2 School of Exercise Science, Physical and Health Education, University of Victoria, STN CSC, PO Box 1700, Victoria, BC V8W 2Y2 Canada; 3 School of Psychology, Sport and Health Sciences, University of Portsmouth, Cambridge Road, Portsmouth, Hampshire PO1 2ER UK; 4 Alliance for Research in Exercise Nutrition and Activity, Allied Health and Human Performance, University of South Australia, GPO Box 2471, Adelaide, SA 5001 Australia

## Abstract

Professional standards are used to inform and measure educational programs and individual credentialling of many professions, including those in allied health. The objective of this project was to develop consensus on international professional standards for clinical exercise physiologists. The project employed an e-Delphi approach to build a consensus, being guided by a steering committee and an expert working group. The expert working group reviewed the elements and the steering committee overviewed the results of each round. The expert working group were invited to consider whether elements should be included or excluded and, if to be included, whether these should be core or non-core. The wording of the elements was also checked for accuracy. To be included in the end professional standards and as core, each element required 80% consensus from the expert working group. The e-Delphi process found consensus on four overarching professional standard domains for clinical exercise physiology: professional practice; foundational knowledge; assessment and client management; and case formulation and design and delivery of evidence-based interventions. Consensus was also achieved on individual elements. All bar two of the elements were accepted as core, with these two both being included in the professional standards since they were close to meeting the 80% consensus threshold and because they are reflected in the clinical exercise physiology professional standards where this profession is established. The international professional standards consensus has been achieved with the aim of supporting the development of a standardised approach to international education and practice in clinical exercise physiology.

## Key Points

**Table:** 

International professional standards for clinical exercise physiology practice have been created.	
These professional standards are intended to guide education and practice of clinical exercise physiology internationally.	
The clinical exercise physiology standard domains include professional practice, foundational knowledge, assessment and client management, and case formulation and design and delivery of exercise-based interventions.	

## Introduction

Participation in exercise is widely recognised as a key aspect of preventing and managing a plethora of health conditions [1]. The role of exercise has long been acknowledged in clinical guidelines for disease management of some conditions including those involving the cardiovascular, metabolic and musculoskeletal systems [2]. Research into other areas has rapidly increased in recent decades, leading to growing formal acknowledgement in a range of other conditions including neurological diseases, mental illness and cancer [3]. This has, in turn, prompted greater attention to the role of health professionals in delivering therapeutic exercise to people and the education and training required to do this safely and effectively [4, 5].

Specific qualifications in the delivery of therapeutic exercise as a primary modality have emerged or grown in prominence in various countries around the world; the Clinical Exercise Physiologist is an example of this with nationally recognised qualifications established in a number of countries [6]. The profession exists in this context in Australia, Canada, New Zealand, the United Kingdom and the United States of America, and is evident in an emergent capacity in other countries [7–9]. The intent of these national level qualifications is clear and largely consistent, being to safely and effectively prescribe and deliver exercise interventions to promote health in people at risk of or with established disease or injury [10].

A clear description of professional standards, defining the knowledge and abilities that underpin safe and competent practice, is commonplace in professional and para-professional qualifications [11]. This is especially so in the health professions where these professional standards commonly underpin the accreditation of higher education providers and certification or registration of individual practitioners in Australia [12], the United Kingdom [13] and the United States of America [14]. This is also the case for clinical exercise physiology whereby these standards define an established health professional in each of these countries: Australia (Exercise & Sport Science Australia [15], 2026); Canada (Canadian Society of Exercise Physiology [16], 2025); New Zealand (Clinical Exercise Physiology New Zealand [17], 2025); the USA (American College of Sports Medicine [18], 2025) and the UK (Clinical Exercise Physiology UK [19], 2025). In many professional areas, including in health, there is recognition of the value of creating global level professional standards that help grow professions in countries where they have not existed and improve international portability of standards [11, 20, 21]. In addition, there is awareness that when professional standards are developed there is a need to recognise the richness and magnitude of global society and the many cultures in which the standards will be ultimately be applied [11, 22]. The aim of this consensus process was to build on recent work to map standards across established countries and create expert consensus-based international clinical exercise physiology professional standards.

## Methods

### Design

This consensus statement project used a modified e-Delphi model, combining online surveys distributed via email and focus groups conducted via videoconference to establish an expert consensus. The participants (expert working group) were asked their opinions on what attributes should be included in entry-level professional standards for clinical exercise physiology practice to inform the global standards. The professional standards were made up of 'Professional Standard Domains', which describe the overarching themes in the professional standards, and 'Elements' that list discrete descriptors of attributes in the standard domain. Ethics approval to conduct this project was obtained from Griffith University Human Research Ethics Committee (GU Ref No: 2024/066).

### Steering Committee

A steering committee was established prior to the commencement of this research project to provide oversight of the research. Members of the steering committee (*n* = 4) had all previously been involved in the development of clinical exercise physiology professional standards in their respective countries, served as members of professional associations whose membership included clinical exercise physiology professionals and had extensive experience teaching clinical exercise physiology professional competencies in university level education programs. Each member of the steering committee was a board director of the International Confederation of Sport and Exercise Science Practice (ICSESP) which is committed to the promotion of international professional standards for sport and exercise science professionals [6].

### Participants

A panel of experts, referred to as the ‘Expert Working Group,’ was recruited for the consensus development process based on content-specific expertise and experience as outlined below and aligned with themes consistent with those established in the literature as ‘expert’ [23, 24]. Recruitment of the expert working group occurred internationally via direct and indirect methods. The direct recruitment process involved members of the ICSESP utilising their networks to identify and invite individuals with recognised knowledge of and experience in the field of clinical exercise physiology. Indirect recruitment involved a process whereby individuals could self-nominate their interest to be in the working group. All direct and indirect working group nominations self-selected for the working group based on meeting the minimum requirement of an ‘expert’ in the field of clinical exercise physiology professional standards. For this project an expert was defined as someone who meet at least one and ideally more than one of the following criteria:

- A university qualification and extensive experience in applying the knowledge and skills of clinical exercise physiology practice (greater than 10 years)
- Experience in developing or reviewing clinical exercise physiology professional standards
- Development of a clinical exercise physiology curriculum across multiple domains of knowledge and skills at the postgraduate university level (or equivalent, i.e. 4-year bachelor degree)

Consistent with recognised features of e-Delphi studies [25, 26], the target expert working group sample size was 15–30, with representation sought from all countries with existing clinical exercise physiology national standards; those countries where clinical exercise physiology national standards were an active aspiration; and all countries where there was an existing clinical exercise physiology workforce. No more than two representatives from any individual country were permitted, except for the USA which was allowed a maximum of three representatives, owing to the variety of professional associations that represent clinical exercise physiology practice.

Expert working group members were provided with a Participant Information Sheet and a Consent to Participate Form at the commencement of the project and responses to all surveys remained anonymous and confidential and only accessible to the steering committee. Each item was put through up to two rounds of voting. The specific stages in the consensus process were as follows: initial voting of previously established standards (1a), identification of additional relevant elements (1b), review of non-consensus items and addition of rationale for decision (2a), voting on additional elements (2b), consensus meeting and final voting on all elements (3), final steering committee deliberations and an external stakeholder review. A consensus threshold was defined a priori as 80% of expert working group members voting for the importance of any individual item above a 7 on a 9-point Likert scale, in accordance with previously published approaches in this field [27, 28]. In addition to the Likert rating, expert working group members were asked to also consider if they viewed an element as ‘core’ or should still be included, but as ‘non-core.’ This allowed them to consider an element to be important for practice as a clinical exercise physiologist but not necessarily essential in the global context for any reason. For this purpose, ‘core’ was defined as ‘cornerstone or an essential part of the professional standard domain of professional practice in all countries and regions’ and ‘non-core’ was defined as ‘an important but not cornerstone or an essential part of the professional standard domain of professional practice in all countries and regions.’ Elements that reached consensus were not voted on in subsequent rounds. One author (NR) thematically coded the survey free text responses in rounds 1b and 2a. All online surveys were conducted using Microsoft Forms with access links sent to the expert working group members via email. Meetings of the expert working group were completed online using the Microsoft Teams videoconference platform.

### Round 1a Survey: Initial Ranking of Elements from Audit

**Fig. 1:**
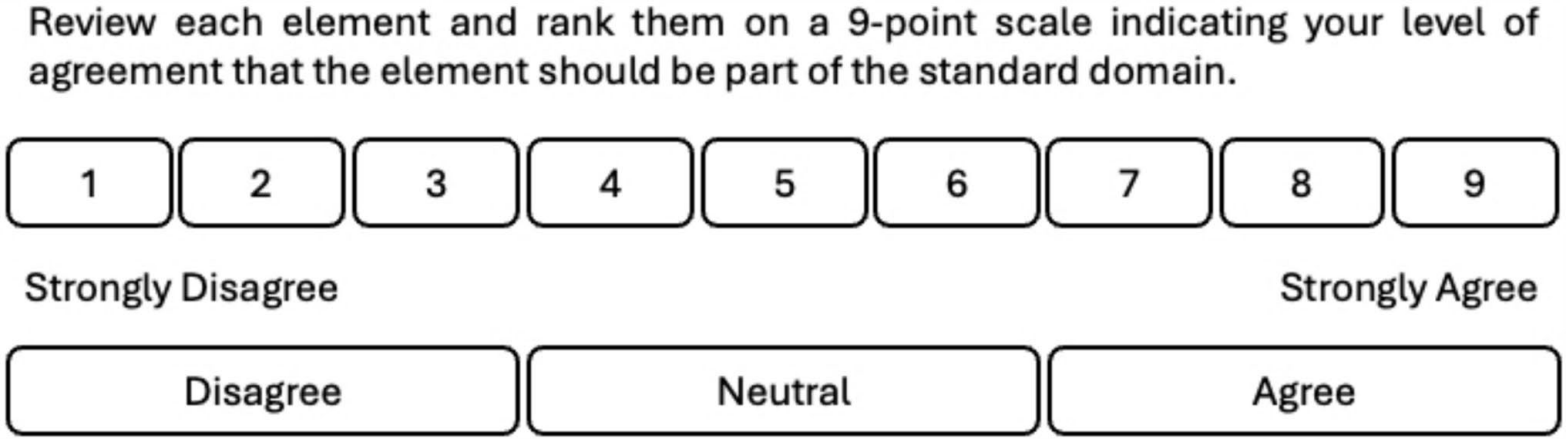
Nine-point Likert scale to rate agreement on the element being included as part of the standard domain

Prior to opening the round 1a survey, expert working group members were asked to review the list of standards identified in an audit of existing clinical exercise physiology standards internationally [7]. The expert working group members were emailed links to four online surveys, one for each of the professional standard domains identified in the audit findings (Professional Practice, Foundational Knowledge, Assessment & Client Management and Design & Delivery of Exercise-Based Interventions). Expert working group members were asked to independently review the elements within the standard domain and rank them on a 9-point Likert scale (see Fig. 1), indicating their level of agreement that the element should be part of the international professional standards.

**Fig. 2:**
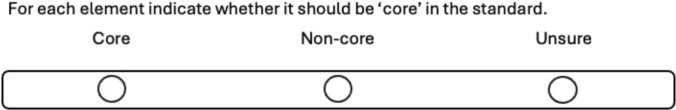
Rating of element as core, non-core or unsure

In Part 2 of the same survey, expert working group members were asked to rate each of the elements as core, non-core, or unsure (Fig. 2).

A ranking of ‘core’ was reflective of the expert working group members indicating that the element should be part of the international professional standards, ‘non-core’ was reflective of the expert working group members indicating that the element should not be part of the international professional standards, and an ‘unsure’ ranking was reflective of the expert working group members indicating that it was unclear to them if the element should be part of the international professional standards.

### Round 1b Survey: Identification of Additional Elements

In round 1b, expert working group members were provided with the list of elements identified in the audit in each of the professional standard domains and asked to consider if there were any elements not yet included that should be. Expert working group members were encouraged not to identify any regionally specific elements (i.e. those elements that would only be relevant at a local level), but rather elements that would have global relevance. A single online survey was used to share the list of existing elements and for collecting expert working group member responses. The survey responses collected were thematically clustered and a draft element was agreed by the steering committee in preparation for round 2b.

### Round 2a Survey: Ranking Rationale

In round 2a, expert working group members were provided with the list of elements from round 1a that did not reach consensus for being part of the international professional standards and/or for not being identified as core. Expert working group members were asked to describe why they rated the element on the 9-point Likert scale the way they did, and/or why they rated the element as core, non-core, or unsure. A single online survey was used to share the list of elements for collecting expert working group member responses.

### Round 2b Survey: Ranking of Additional Elements

In round 2b, expert working group members were provided with the list of new elements identified in round 1b and asked to rank them on a 9-point Likert scale indicating their level of agreement that the element should be part of the international professional standards, and if it should be core, non-core or unsure. To assist the expert working group members in ranking the element they were provided with a list of elements across the four professional standard domains that were considered in the round 1a survey. As occurred in the round 1a survey, responses to each of the elements were summed to determine the level of consensus for each element in relation to agreement that the element should be part of the international professional standards, and if so, should it be core or non-core. Consensus was set as ≥ 80% of agree (scores of 7–9) and core (scores of core and unsure).

### Round 3 Survey: Consensus Meeting and Re-voting of All Items

In round 3, expert working group members attended an online focus group to discuss the elements that had not met the consensus threshold to be part of the international professional standards and/or core in round 1b and 2b. Group discussion was followed by expert working group members using an online survey to indicate their agreement on a 9-point Likert scale that the element should be part of the international professional standards, and if it should be core or non-core. In addition, during the discussion groups the expert working group members were asked to declare if they wanted to revise the wording of any of the elements (including those elements that had achieved consensus and those that had not). Prior to the focus group, expert working group members were provided with the round 2a survey responses to aid their preparation. Three discussion groups were scheduled over a 24-h period, 6 h apart to ensure expert working group members in various time zones could attend. Expert working group members were invited to attend one of the focus groups.

### Steering Committee Recommendations

The steering committee reviewed the consensus outcomes captured in the round 1a and 3 surveys for each of the original and additional elements. The steering committee was also presented with expert working group member feedback on alternative wording for the elements. Using open discussion and collective decision-making, the steering committee subsequently agreed upon the first draft of the international clinical exercise physiology professional standards. At this stage, the steering committee discussed the precise wording of each element and whether elements receiving < 80% expert working group consensus for being included should still be included, given their importance to international practice as a professional clinical exercise physiologist, and no further decisions were required.

#### External Stakeholder Feedback

External stakeholder feedback was sought on the draft international clinical exercise physiology professional standards over a 6-week period. Recruitment of external stakeholders was both targeted (i.e. direct invitations to people with similar experience to the working group) and broad (i.e. email invitations sent to founding partners of the ICSESP to disseminate to their membership). Background on the work completed (including preface, project governance, project contributors, and references) and the draft professional standards was made available via the ICSESP website. A link to an online Microsoft Forms survey was provided on the website for feedback to be provided. Steering committee members forwarded website links to individuals as part of the targeted recruitment.

Comments from external stakeholders were analysed using an inductive framework approach to identify naturally emerging themes. The initial themes were identified by N.R. and K.L. and then reviewed and agreed upon by the other co-authors, after which the remaining comments were systematically coded [29]. This summary was presented to the steering committee for review. Using open discussion and collective decision-making, the committee decided if the feedback warranted a modification of the draft professional standards. A revised version of the draft professional standards was developed.

The expert working group were emailed a copy of the updated draft of the international clinical exercise physiology professional standards and asked to provide feedback. A total of seven email responses were received from the expert working group which were summarised and presented to the steering committee for review. Using open discussion and collective decision-making, the steering committee decided if the feedback warranted a modification to the draft professional standards. A final version of the draft professional standards was developed.

## Results

### Expert Working Group

A total of 21 individuals were recruited to contribute to the consensus process from a broad representation of geographic locations (Table 1). Of those recruited, 19 completed round 1a (response rate 90%), 13 completed round 1b (response rate 62%), 15 completed round 2a/b (response rate 71%) and 14 completed round 3 (response rate 67%). Expert working group members reached consensus that 20 elements be included in the international clinical exercise physiology professional standard.

**Table 1:** Expert working group demographics

Post-secondary education					
Bachelor’s degree	1				
Master’s degree	5				
Doctoral degree	15				
Level of standards expertise					
Curriculum design	15				
Clinical practice	11				
Policy	4				

	Round 1a	Round 1b	Round 2a	Round 2b	Round 3
Total number of surveys completed (*n*)	19	13	15	15	14
Completion Rate (%)	90	62	71	71	67
Country of origin (*n*/invited)					
Australia	2/2	2/2	2/2	2/2	2/2
Canada	2/2	1/2	2/2	2/2	2/2
China	1/1	1/1	1/1	1/1	0/1
Croatia	0/1	0/1	1/1	1/1	0/1
Denmark	0/1	0/1	1/1	1/1	1/1
Italy	1/1	0/1	0/1	0/1	0/1
Ireland	1/1	0/1	1/1	1/1	1/1
New Zealand	2/2	2/2	1/2	1/2	2/2
Poland	1/1	1/1	0/1	0/1	1/1
Portugal	2/2	2/2	0/2	0/2	1/2
South Africa	1/1	0/1	0/1	0/1	0/1
Sweden	1/1	1/1	1/1	1/1	0/1
United Kingdom	2/2	1/2	2/2	2/2	1/2
United States of America	3/3	2/3	3/3	3/3	3/3
Post-secondary education					
Bachelor’s degree	1	1	1	1	1
Master’s degree	5	2	4	4	3
Doctoral degree	13	10	10	10	10
Level of standards expertise					
Curriculum design	13	8	11	11	10
Clinical practice	11	9	9	9	8
Policy	4	3	2	2	4

### Expert Working Group Consensus on Elements That Should Be Included

In round 1a, consensus was reached on 18 of 21 elements across the four professional domains that were identified from an audit of clinical exercise physiology professional standards (Table 2).

**Table 2:** Respondent agreement on inclusion and core/non-core of professional standards for clinical exercise physiology practice identified from the audit

Element	Round 1a		Round 3		Round 1a		Round 3	
	Agree (%)	Disagree (%)	Agree (%)	Disagree (%)	Core (%)	Non-core (%)	Core (%)	Non-core (%)
Professional standard domain 1—professional practice								
1.1 Practice within Scope of Practice, Code of Conduct and Ethical Practice	95	5			100	0		
1.2 Practice in accordance with legislation, regulations and standards^a^	95	5			100	0		
1.3 Develop effective, concise, respectful and informative clinical documentation and reports	100	0			74	26	100	0
1.4 Practice in a culturally safe, inclusive, sensitive and respectful way and according to person-centred care principles^b^	100	0			95	5		
1.5 Practice collaboratively with other professionals^c^	100	0			63	37	100	0
1.6 Demonstrate emergency procedures	84	0			79	21		
1.7 Develop reflective practice^d^	68	32	79	21	37	63	21	79
1.8 Demonstrate leadership and ability to advocate for client access	63	37	36	64	42	58	37	63
Professional standard domain 2—foundational knowledge								
2.1 Integrate foundational knowledge and apply these to inform safe and effective movement, physical activity and exercise-based interventions for individuals and population groups throughout all stages of their life	100	0			100	0		
2.2 Examine principles of biopsychosocial care, value-based care, person-centred care and cultural determinants of health and apply this to promote health and well-being for individual clients and population groups	84	0			79	21	89	11
2.3 Evaluate the effect of commonly prescribed medications, diagnostic procedures, medical, surgical, and other interventions on both resting and exercise-related physiological responses across the full health spectrum	95	5			84	16		
2.4 Evaluate and apply contextual learning principles and behaviour change strategies to improve health outcomes, increase engagement, motivation and adherence and empower self-management of health conditions​	95	0			84	16		
2.5 Explain national, state and compensable scheme frameworks across the health care, aged care and disability sectors, and the requirements for CEPs working in these settings	63	11	68	21	32	68	32	68
Professional standard domain 3—assessment and client management								
3.1 Distinguish, record, report, and appropriately action changing risk factors and adverse signs and symptoms that may arise before, during, and after assessments and interventions	100	0			95	5		
3.2 Distinguish and communicate appropriate client support to effect service delivery including onward referral and using various modalities to communicate​	100	0			84	16		
3.3 Formulate appropriate screening processes to evaluate and stratify risk^e^	100	0			100	0		
Professional standard domain 4—design and delivery of evidence-based interventions								
4.1 Formulate evidence-based exercise prescription, interventions and recommendations that address health and treatment related client needs^f^	100	0			100	0		
4.2 Design, prescribe, deliver and monitor safe and effective movement, physical activity and evidence-based interventions for clients with complex presentations​	89	0			95	5		
4.3 Formulate and apply strategies to manage risk, evaluate progress and adapt recommendations and interventions	100	0			100	0		
4.4 Create and apply communication strategies to educate and engage clients	89	11			84	16		
4.5 Formulate strategies during treatment to empower clients​	84	11			63	37	74	26

^a^This element from the audit was excluded from the final draft of the professional standards in favour of the revised wording (Table 3—A.1.9)

^b^This element was combined with another element added by experts (Table 3—A.1.11)

^c^This element from the audit was excluded from the final draft of the professional standards in favour of the revised wording (Table 3—A.1.10)

^d^The research team elected to keep this element from the audit in the final draft of the professional standards despite not reaching consensus

^e^This element from the audit was excluded from the final draft of the professional standards in favour of the revised wording (Table 3—A.3.4)

^f^This element from the audit was excluded from the final draft of the professional standards in favour of the revised wording (Table 3—A.4.6)

Expert working group members were then asked via open text boxes in the round 1b survey to propose elements that should be added. Eight additional elements were proposed and voted on in round 2b with five reaching a consensus (Table 3).

**Table 3:** Respondent agreement on inclusion and core/non-core for additions to professional standards for clinical exercise physiology practice identified from the audit

Element	Round 2b		Round 3		Round 2b		Round 3	
	Agree (%)	Disagree (%)	Agree (%)	Disagree (%)	Core (%)	Non-core (%)	Core (%)	Non-core (%)
Professional standard domain 1—professional practice								
A.1.9 Practice in accordance with legislation, regulations and standards, and respect the privacy and confidentiality of all personal information^a^	88	6			94	19		
A.1.10 Practice collaboratively with other professional and promote onward referral pathways as indicated^b^	88	6			81	19		
A.1.11 Practice consistent with equity, diversity and inclusion^c^	75	13	81	0	81	19		
Professional standard domain 2—foundational knowledge								
A.2.7 Integrate foundational knowledge (including functional anatomy, human anatomy, human physiology, exercise physiology, exercise prescription and delivery, biomechanics, growth and development, health and exercise assessment, motor learning and control, nutrition, physical activity and health, psychology of health and exercise, and research methods and data analysis) and apply these to inform safe and effective movement, physical activity and exercise-based interventions for individuals and population groups throughout all stages of their life^d^	69	31			81	19		
Professional standard domain 3—assessment and client management								
A.3.4 Formulate appropriate screening processes to evaluate and stratify risk, and the formulation, implementation and evaluation of exercise prescription to realize a client’s physical activity, exercise, health and/or functional performance goals^e^	81	13			88	12		
A.3.5 Determine absolute and relative contraindications to exercise and utilize a variety of evidence-based standardized and individualized measurements, including patient reported outcome measurements	94	6			94	6		
Professional standard domain 4—design and delivery of evidence-based interventions								
A.4.6 Formulate meaningful and goal-orientated evidence-based exercise prescription, interventions and recommendations that address health and treatment related client needs^f^	100	0			100	0		
A.4.7 Create and apply contemporary and emerging communication strategies to educate and engage clients relative to their learning style, health literacy and environment^g^	69	13			69	31		

^a^This proposed element was chosen in place of one from the audit (Table 2—1.2)

^b^This proposed element was chosen in place of one from the audit (Table 2—1.5)

^c^This element was combined with another element from the audit (Table 2—1.4)

^d^This proposed element was excluded from the final draft of the professional standards in favour of the original element from the audit (Table 2—2.1)

^e^This proposed element was chosen in place of one from the audit (Table 2—3.3)

^f^This proposed element was chosen in place of one from the audit (Table 2—4.1)

^g^This proposed element was excluded from the final draft of the professional standards in favour of the original element from the audit (Table 2—4.4)

Prior to voting in round 3, an online consensus meeting was arranged to discuss elements that still had not reached consensus (three original elements and three proposed elements). A total of 14 expert working group members attended the online discussion groups (six in online discussion meeting #1, three in online discussion meeting #2 and five in online discussion meeting #3). At the conclusion of these online meetings, expert working group members voted on all elements that had not yet reached consensus. None of the three original items reached consensus in this round (Table 2), whereas one of the three additional/modified elements did (Table 3).

These discussions, along with the resulting round 3 vote, led to 13 out of 21 elements from the original audit being included in the final draft with only minor modifications. One element from the audit did not reach consensus but was still included (discussed *in *Sect. 3.4 below) while the other two elements that did not reach a consensus were excluded (Table 2).

Regarding the additional/modified elements, five out of eight reached consensus and were included in the final draft. Specifically, four elements (Table 3—A.1.9, A.1.10, A.3.4, A.4.6) were chosen in place of original elements from the audit (Table 2—1.2, 1.5, 3.3, 4.1) and one was added to the Assessment and Client Management Domain (Table 3—A.3.5). One additional/modified element (Table 3—A.1.11) was combined with an original element (Table 2—1.4) on the basis of external feedback. Finally, two elements did not reach consensus and so were excluded (Table 3).

### Expert Working Group Consensus on Elements That Should Be Core

After two rounds of voting (round 1a and round 3), 16/21 elements from the audit reached consensus for being a core element (Table 2). In terms of the eight proposed elements, seven reached a consensus for being a core standard in round 2b. As described in Sect. 3.2, redundancies were noted between the original elements and proposed elements resulting in some being excluded from the final document. Thus, of the 20 elements included in the final draft, 18 reached a consensus for being a core element.

### Steering Committee Recommendations

The steering committee reconvened after round 3 and considered the results of the e-Delphi data to finalize recommendations for the Draft International Clinical Exercise Physiology Professional Standards. Decisions occurred by consensus at this meeting. The steering committee’s discussion considered elements 1.7 ‘*Develop reflective practices*’ and 4.5 *‘Formulate strategies during treatment to enable clients in self-management'* which had not reached consensus for inclusion in round 3 (79%). The committee decided that as reflective practice and promoting self-management are elements that are included within the professional standards documents of other health care professions in several regions, they would be included in the draft standards [30–32].

Changes to the wording of four other elements as proposed by participants in round 3 was accepted by the steering committee (Table 4). At this stage, the committee completed a final review of all the included elements and prepared the Draft International Clinical Exercise Physiology Professional Standards for external feedback.

**Table 4:** Finalized wording for the International Clinical Exercise Physiology Professional Standards

Final element	Notes
Professional standard domain 1—professional practice	
1.1 Practice within Scope of Practice, Code of Conduct and Ethical Practice	
1.2 Practice in accordance with legislation, regulations and standards, and respect the privacy and confidentiality of all personal information	Experts selected the modified element (Table 3—A.1.9) over the original element (Table 2—1.2)
1.3 Develop effective, concise, respectful and informative clinical documentation and reports	
1.4 Practice in a culturally safe, sensitive and respectful way consistent with principles of equity, diversity and inclusion, and according to person-centred care principles	Two elements were combined on the basis of external feedback (Table 2—1.4 and Table 3—A1.11)
1.5 Practice collaboratively with other professionals and promote onward referral pathways as indicated	Experts selected the modified element (Table 3—A.1.10) over the original element (Table 2—1.5)
1.6 Identify potential client risks and demonstrate effective risk management and mitigation strategies	Wording of the original element (Table 2—1.6) was revised based on external feedback
*1.7 Develop reflective practice	Steering committee elected to keep this element despite it not reaching consensus (Table 2—1.7)
*Note: Reflective practice can include a range of individual or group activities and initiatives undertaken by a Clinical Exercise Physiologist at any stage across the learner to experienced practitioner continuum	This note was added to the Professional Practice domain based on external feedback
Professional standard domain 2—foundational knowledge	
2.1 Integrate foundational knowledge and apply these to inform safe and effective movement, physical activity and exercise-based interventions for individuals and population groups throughout all stages of their life	
2.2 Examine principles of biopsychosocial care, value-based care, person-centred care and cultural determinants of health and apply this to promote health and well-being for individual clients and population groups	
2.3 Evaluate the effect of commonly prescribed medications, diagnostic procedures, medical, surgical, and other interventions on both resting and exercise-related physiological responses and adaptations across the full health spectrum	‘…*and adaptations…*’ was added to the original wording of the element (Table 2—2.3) based on external feedback
2.4 Evaluate and apply contextual learning principles and behaviour change strategies to improve health outcomes, increase engagement, motivation and adherence and empower self-management of health conditions​	
NOTE: A clinical Exercise Physiologist’s foundational knowledge will ideally include the following sub-disciplines: human anatomy, functional anatomy, human physiology, exercise physiology, exercise prescription and delivery for clinical populations, biomechanics, growth and development, health and exercise assessment, motor learning and control, nutrition, physical activity and health, psychology of health and exercise, and research methods and data analysis	This note was added to the Foundational Knowledge domain based on external feedback
Professional standard domain 3—assessment and client management	
3.1 Distinguish, record, report and appropriately action changing risk factors and adverse signs and symptoms that may arise before, during, and after assessments and interventions	
3.2 Develop client-centred methods to provide effective service delivery using various communication methods, including referral to relevant health care professionals	Wording of element 3.2 (Table 2) was revised based on participant feedback
3.3 Formulate appropriate screening processes to evaluate and stratify risk, and the formulation, implementation and evaluation of exercise prescription to realize a client’s physical activity, exercise, health and/or functional performance goals	Experts selected the modified element (Table 3—A.3.4) over the original element (Table 2—3.3)
3.4 Determine absolute and relative contraindications to exercise and utilize a variety of evidence-based standardized and individualized measurements, including patient reported outcome measurements	This element reached consensus in round 2b (see Table 3—A.3.5) and so was added to this standard domain
Professional standard domain 4—design and delivery of evidence-based interventions	
4.1 Formulate meaningful and goal-orientated evidence-based exercise prescription, interventions and recommendations that address health and treatment related client needs	Experts selected the modified element (Table 3—A.4.6) over the original element (Table 2—4.1)
4.2 Design, prescribe, deliver and monitor safe and effective movement, physical activity and evidence-based interventions for clients with complex presentations​	
4.3 Formulate and apply strategies to manage risk, evaluate progress and adapt recommendations and interventions	
4.4 Create and apply communication strategies to educate and engage clients	
*4.5 Formulate strategies during treatment to enable clients in self-management	Wording of the original element (Table 2—4.5) was revised based on participant feedback
*Note: Being evidence-based and evidence-informed are both identified as being critical to making informed clinical decisions	This note was added to the Design and delivery of evidence-based interventions domain based on external feedback

### External Stakeholder Feedback

External stakeholder feedback on the Draft International Clinical Exercise Physiology Professional Standards was received from 27 individuals from seven countries (Table 5). A total of 63% of respondents provided detailed feedback with actionable changes that could be made to the standards. Overall, 59% gave positive reviews of the overall document, suggesting their enthusiasm for ‘universal’ standards and the creation of a global ‘benchmark.’ Feedback on areas that could be improved included lack of detail within some of the domains, redundant elements and language. In terms of specific feedback, several themes emerged including lack of detail within some of the standard domains, redundant elements and language concerns. Table 6 provides a summary of the themes and sub-themes with representative quotes that add insight or meaningfulness.

**Table 5:** External stakeholder demographics

Country of origin	
Australia	5
Canada	10
China	1
New Zealand	2
Norway	1
UK	2
USA	6
Post-secondary education	
Bachelor’s degree	6
Master’s degree	10
Doctoral degree	11
Level of standards expertise	
Curriculum design	13
Clinical practice	17
Policy	3

**Table 6:** Summary of external feedback on the Draft International CEP Professional Standards

Themes	Sub-themes	Example quotes
Lack of detail	Domain 1—Professional Practice	‘*In the professional practice section I would include 'areas of knowledge' in the first core element. E.g. Practice within scope of practice, areas of knowledge, code of conduct and ethical practice. I suggest this because although a task is in my scope of practice, if it is not an area that I have practiced in for the last 20 years, I am going to up skill before engaging in the task*.’(6) ‘*can you add "ethical practice as defined by local government, organizational practice, *etc*.". These types of laws or ethical policies will vary depending on location*.’ (10)
	Domain 1—Professional Practice (specifically; emergency procedures)	‘*Demonstrate emergency procedures. Needs some details. E.g. Implements appropriate risk planning and demonstrates effective risk management strategies including emergency responses procedures*.’ (4) ‘*I see a lack of "emergency procedures" but perhaps this falls under the risk mitigation / management components*.’(5) ‘*While noted in the professional practice domain, there appears to be a lack of focus on the client in this section beyond risk mitigation and management.’* (23)
	Domain 2—Foundational Knowledge	‘*In the Standard Domain: Foundational Knowledge- the first point may be too generic, providing some more scope or at least examples of what would fall within the Foundational knowledge may support ensuring measurable knowledge competencies can be evaluated in programs.’* (5) *‘In Foundational Knowledge domain: consider adjusting to "social and cultural determinants of health.’* (22) *‘Foundational knowledge: While the domain sets a strong knowledge base, it lacks specificity regarding the clinical settings or populations a CEP should be equipped to work with. Explicit terms related to diverse clinical populations are critical. The current reference is too general (i.e., improving health, fitness, well-being, and performance across various populations). This element should clearly distinguish CEPs from personal trainers and exercise scientists. The current statement could apply to personal trainers or exercise scientists here in Aus, indicating a major gap that needs addressing to ensure consistent, high-quality practice globally.’ (23)*
Redundancies	Cultural safety & equity	‘*Professional practice—4th and 7th line seem quite similar. Is there a way to merge those line together? (Ex. Practice consistently in a culturally safe, inclusive, sensitive, diverse, equitable and respectful way in accordance with person-centered care principles.)’* (3) ‘*Core elements 4 and 7 are similar and should be combined.’* (10)
	Communication	‘*Design & Delivery of EB Interventions- The last core point of communication is somewhat repetitive to what was above in Client Management but if this is meant as a different part of communication (e.g. to support EB interventions) then it may be helpful to delineate how this is meant (to support adherence? to communicate aspects of exercise prescription?)*’ (5)
Language		‘*Suggest providing some (actionable/descriptive) detail of each Core/Non-Core Elements in support of the Element Statements*’ (13) ‘*Clearly defining the purpose and scope of the standards will help users understand the rationale and applicability of each section.*’ (18) ‘*Formulate strategies during treatment to enable clients" from the design and delivery of EB interventions appears to be an incomplete sentence. Enable clients to*?’ (21)

### Final International Clinical Exercise Physiology Professional Standards

The steering committee met after receiving a summary of the external feedback. Each piece of feedback was considered with several changes made to the Draft International Clinical Exercise Physiology Professional Standards. The result was an International Clinical Exercise Physiology Professional Standards document that included 20 elements across four professional standard domains (Table 4).

## Discussion

The aim of this project was to undertake a methodologically rigorous approach to defining the core professional standards that underpin the practice of clinical exercise physiology across the world. A number of countries have established national clinical exercise physiology professional standards, as has been reported previously [7], but the development of international professional standards using expert consensus is a novel contribution. This project presents a robust approach to consensus development for the purpose of defining professional standards. The application of rigorous consensus development approaches goes beyond what is typically used for professional standards development at the national level, or indeed across other professions. The findings of this project herald a new chapter for the clinical exercise physiology profession, facilitating growth and recognition across the world and better alignment and collaboration between countries. With the International Clinical Exercise Physiology Professional Standards to build upon, a next step may be an international accreditation framework for clinical exercise physiology akin to other health professions. The ICSESP organisation is ideally placed to support this work and model this on the success of other organisations.

Importantly, these professional standards ensured representation from countries beyond those that currently have an established clinical exercise physiology profession. The inclusion of countries where the clinical exercise physiology was an active aspiration allowed these standards to represent the broader global and anticipated future work of the clinical exercise physiology profession, rather than simply reflect the status quo. A total of 21 experts representing 14 countries and five continents participated in this project which conferred a strong level of global engagement in the production of the standards. While not all expert working group members contributed to each round of the project, there were at least 13 expert working group members across all rounds which is more than the minimum number expected to produce reliable consensus findings [33]. External stakeholder feedback on the initial draft standards was received from 27 individuals across six countries. The purposeful consideration of this feedback further contributed to the quality of the final set of standards.

The audit findings of existing clinical exercise physiology accreditation standards [7] across the foundation member organisations of the ICSESP were used as the starting point for this project, and which consistent with previous approaches [23, 24]. This provided the expert working group with professional standard domains and individual elements to base their consensus deliberations on and proved highly beneficial in supporting the progress of this project. These original professional standards have been developed from established nations, with American College of Sports Medicine (ACSM), in particular, taking a job task analysis approach to defining their clinical exercise physiology professional standards to ensure that these are occupationally relevant [7]. The UK’s approach to developing their professional standards was largely adapted from the Exercise & Sports Science Australia’s (ESSA) professional standards, which were less prescriptive than their initial iteration yet more detailed than their final iteration, with the chosen mid-iteration being suitable to not only guide the high-level professional standards but also to be used as a curriculum development guide [10]. These standards are based on what a clinical exercise physiology professional is expected to be able to do in the workplace [7]. After two rounds of voting, incorporation of external feedback and deliberations of the steering committee, 14 of the original 21 audit elements were included in the final set of standards, with four of these undergoing minor wording revisions. Four proposed/modified elements replaced similarly stated original elements and another element that was not previously identified in the audit findings was added to the final standards. A total of 18 of the 20 elements were recognised as being core to the professional standards and an essential part of the competency of clinical exercise physiologists, with two elements instead being recognised as highly desirable practice behaviours. Setting the consensus threshold for ranking of elements for inclusion and core at 80% in each round of the project contributed to the quality and overall level of agreement with the final standards. Elements ‘1.7 Develop reflective practice’ and ‘4.5 Formulate strategies during treatment to enable clients in self-management’ were deemed by the steering committee to be essential functions of the role of a healthcare professional, with element 4.5 being reworded so that performing empowering behaviour change techniques are known as core. In the broader context of the expert working group it is possible that in the ‘physiology world’ such knowledge and skills are not seen as core, but as professional healthcare practitioners, these are both necessary for the benefit of the professional, their profession and their service users for whom they are responsible [34]. These world-leading professional standards need to set the standards rather than leave them to choice.

Interestingly, many healthcare professions are harmonising education requirements and standardising credentials globally [35–37]. This shift is being motivated by the need to meet an ever-increasing global demand for high quality healthcare, and an imperative to educate providers of healthcare to respond effectively to current and emerging challenges. The standards produced in this project support the clinical exercise physiology profession at an international level to provide consistency in knowledge and skills in the industry and will similarly position the profession to align with the global shift being seen in other professions (International Confederation of Dietetic Associations [ICDA], World Confederation for Physical Therapy [WCPT] [now known as World Physiotherapy], World Federation of Occupational Therapists [WFOT]). In addition, international clinical exercise physiology professional standards hold the promise of supporting increased homogeneity between national accreditation frameworks which will encourage mutual recognition of qualifications and the international mobility of the clinical exercise physiology workforce.

The existence of International Clinical Exercise Physiology Professional Standards provide a framework for national organisations with established clinical exercise physiology accreditation and credentialling to map their clinical exercise physiology standards against. This will give organisations confidence that their standards meet local needs and those of the profession globally. In countries and jurisdictions where clinical exercise physiology national organisations remain an aspiration, the standards represent a beacon that will help them navigate towards assembling quality assurance frameworks to support the development of a highly capable and competent national clinical exercise physiology workforce. These standards should be used to guide minimum international thresholds for what a clinical exercise physiology professional can do, including a scope of practice, and to develop degree programs that include the necessary education, technical skill development and clinical practice-based learning exposure to emulate these standards. Numerous countries have a version of this clinical track, sometimes referred to as clinical exercise physiology, therapeutic exercise or similar variations. These standards can unify such professional nomenclature into a single recognised professional approach. The authors acknowledge that it may be a long process to become truly global, with differences in the clinical approaches in the sport and exercise sciences and technical requirements for fitness leadership in common healthcare professions [4]. Individual nations require a governing body to guide the integration of these professional standards into their degree programs to ensure quality, such as education and training standards guides to support the translation of these standards into an education pathway.

Nations that currently accredit/certify/register clinical exercise physiologists have accrediting organisations for both educational degrees and for appropriately qualified and experienced individuals, with statutory or non-statutory clinical registers for professionals to maintain their clinical registration through fitness to practise and continuing professional development requirements [10]. For nations without a current registration/accrediting body for clinical exercise physiologists, steering groups for such a profession could collaborate with another national healthcare association to provide the education and standards quality assurance governance procedures to ensure parity with other healthcare professions in those countries [38]. Collaborative practice in healthcare has been shown to deliver improved patient outcomes [39] and increase return on investment in healthcare funding [40]. Having globally synchronised professional standards for clinical exercise physiology lays the foundation for clearer and more effective engagement with international bodies. For example, working with partners such as the WFOT, WCPT and the ICDA can support the development of collaborative care models that can filter down to national level policy and practice.

A key strength of this project is the robust methodological approach to consensus development, including a high threshold for inclusion with the 80% agreement threshold combined with the additional question of core versus non-core rating of the element. A second strength is the inclusion of experts from a wide range of countries with varying levels of progression of the clinical exercise physiology profession. This ensures the standards are valid and useful in contexts where the profession is emerging and not only where it is already established and recognised within the health system and workforce. The representation in the sample of experts and generally high agreement across the elements suggest the results are a valid global set of core standards.

### Limitations

The majority of the experts were derived from the ICSESP international network (*n* = 15, 71%). Whilst these networks were able to enlist a strong field of clinical exercise physiology standards experts from across the world, it is possible that not all relevant expertise and regional context were captured. In particular all but one of the experts were from ‘western’ countries which possibly leaves a lack of cultural, religious and political consideration of ‘eastern’ countries. This level of representation of experts could have limited the depth and breadth, and potentially the transferability, of the standards included in the final version of standards.

## Conclusions

The International Clinical Exercise Physiology Professional Standards described in this project represent the first ever set of international standards for clinical exercise physiology. The 20 elements across four standard domains have been generated by enlisting the contribution of clinical exercise physiology profession experts from around the world using a methodologically rigorous approach to arrive at a consensus. This work and the education, advocacy and policy that can follow it will support the growth of the profession and enhance attempts by the critical health workforce to address the global burden of chronic disease.

## Declarations

### Availability of Data and Material

Data are available from the authors on request.
